# Comprehensive analysis of disulfidptosis-related lncRNA features for prognosis and immune landscape prediction in colorectal cancer

**DOI:** 10.3389/fonc.2023.1287808

**Published:** 2023-12-27

**Authors:** Chengyuan Dong, Yadong Guo, Ping Wang, Shiqi Yin, Xin Ge

**Affiliations:** ^1^ School of Medicine, Anhui University of Science and Technology, Huainan, China; ^2^ Department of Urology, Shanghai Tenth People’s Hospital, School of Medicine, Tongji University, Shanghai, China; ^3^ Department of Clinical Medicine, Shanghai Tenth People’s Hospital, School of Medicine, Tongji University, Shanghai, China

**Keywords:** disulfidptosis, lncRNA, tumor therapies, prognosis, CRC

## Abstract

Disulfidptosis is a novel mechanism underlying actin-cytoskeleton-associated cell death, but its function in colorectal cancer (CRC) is still elusive. In this study, we investigated the potential role of Disulfidptosis-Related Long Non-Coding RNAs (DRLs) as prognostic indicators in CRC. Through transcriptome data from TCGA CRC cases, we identified 44 prognosis-correlated DRLs by Univariate Cox Regression Analysis and observed a differential expression pattern of these DRLs between CRC and normal tissues. Consensus clustering analysis based on DRL expression led to subgroup classification of CRC patients with distinct molecular fingerprints, accompanied by a superior survival outcome in cluster 2. We are encouraged to develop a score model incorporating 12 key DRLs to predict patient outcomes. Notably, this model displayed more reliable accuracy than other predictive indicators since DRLs are intimately related to tumor immune cell infiltration, suggesting a considerable potential of our DRL-score model for tumor therapy. Our data offered a valuable insight into the prognostic significance of DRLs in CRC and broke a new avenue for tumor prognosis prediction.

## 1 Introduction

Colorectal cancer is a highly lethal digestive malignancy, ranking third in incidence and second in cancer-related deaths (1–4). While a comprehensive treatment approach, encompassing both surgery and chemotherapy, has notably enhanced survival rates for colorectal cancer (CRC) patients, 40% continue to grapple with challenges associated with tumor resistance and recurrence (1). However, the invasiveness of the tumor can impact the effectiveness of these treatments, limiting the anticancer effects produced by radiotherapy and chemotherapy (5). Recent studies show that immunotherapy is a promising treatment strategy for advanced CRC with high microsatellite instability, which is still limited to a very small portion of CRC patients (6, 7). Therefore, establishing an effective bio-alarm system is an urgent need for precise detection, diagnosis and treatment guidance for CRC patients.

Disulfidptosis has been identified as a newly discovered mechanism of cell death that involves the actin cytoskeleton within cells (8). Excessive intracellular disulfides can induce disulfide stress, making actin cytoskeleton proteins susceptible to the formation of abnormal disulfide bonds. Ultimately, cell death occurs due to the disruption of the actin network (9, 10). The regulatory genes governing these intricate mechanisms have been meticulously unearthed (8). For instance, inactivation of the genes GYS1, NDUFS1, NDUFA11, NDUBL, and LRPPRC collaborates with glucose starvation to induce cell death (8, 11–15); OXSM contributes to glycogen regulation (16); and NCKAP1, RPN1, SLC3A2, and SLC7A11 collaboratively participate in disulfidptosis regulation (8, 17). Consequently, further investigation into the potential of targeting disulfidptosis as a diagnostic and therapeutic strategy for cancer is warranted.

Long non-coding RNAs (lncRNAs) are non-protein-coding RNA molecules longer than 200 nucleotides that are involved in various biological processes such as cell proliferation, apoptosis, and metastasis via their interactions with proteins, RNAs, and DNAs (18, 19). Studies suggest that lncRNA-CDC6 promotes cell proliferation and metastasis in breast cancer, which is positively correlated with malignant stage (20). Silencing lncRNA-SNHG1 in macrophages inhibits pro-angiogenic and tumor-promoting effects (21). In addition, the lncRNA SH3PXD2A-AS1’s partial expression is associated with tumor size, TNM staging, and metastasis in CRC patients. Moreover, knockdown of SH3PXD2A-AS1 suppresses CRC cell growth, migration and invasion, offering a new target for CRC diagnosis and treatment (20). Taken together, lncRNAs are demonstrated to possess therapeutic potential in early CRC diagnosis and treatment.

This study aimed to assess the expression levels of DRLs in the CRC data set to pursue the objective of constructing a prognostic model to predict individual patient outcomes and facilitate clinical decision-making. Additionally, we performed gene set enrichment analysis (GSEA) and immune infiltration analysis to investigate the mechanisms of DRLs in CRC. Finally, we conducted a preliminary validation of our prediction model according to the differential expression of eight DRLs in CRC cell lines. Our data proved the potential use of this strategy for predicting the prognosis of CRC patients and offers valuable standards for clinical decision-making.

## 2 Materials and methods

### 2.1 Data source and differential expression of disulfidptosis-related genes (DRGs)

TCGA supplied gene transcriptome data, clinical characteristics (n = 458), and mutation details (n = 452) for both normal and CRC samples, serving as the data source for Disulfidptosis-Related genes (DRGs) and their differential expression. Transcription data was processed per kilobase fragment and normalized to one million transcripts. To normalize gene expression levels, log2 (FPKM+1) was utilized. Ten DRGs were identified from previous studies. The Limma software was utilized to detect differentially expressed DRGs with an absolute log2 fold change greater than 1.5 and a false discovery rate (FDR) lower than 0.05.

### 2.2 Visualizing copy number variations (CNV) in colorectal cancer

CNV data was analyzed using Gistic2.0 to identify chromosomal segments with significant amplifications and deletions, comparing CNVs across chromosomal arms. The chromosomal positions of genes were visualized using “RCircos” in R.

### 2.3 Analyzing and clustering DRLs through identification and consensus

DRLs were obtained by performing Pearson correlation analysis on differential expression DRGs, considering a correlation coefficient greater than 0.5 and a P-value less than 0.001. Prognosis was found to be significantly associated with DRLs through univariate Cox regression analysis using the ‘survival’ package. To identify variations in expression of prognosis-associated DRLs between tumor and normal tissues, the Wilcoxon test was employed. We used the “limma” and “corrplot” packages to compute the correlation between PD-L1 expression and DRLs. Unsupervised consensus clustering using the “ConsensusClusterPlus” package identified potential DRL subtypes. We utilized the ‘survival’ and ‘survminer’ packages to conduct survival analysis and compare clinical parameters. Using the ‘CIBERSORT’ package, the proportions of 22 different subtypes of immune cells were estimated between the subgroups. For each patient with CRC, the immune score, stromal score, and tumor purity were acquired using the ‘estimate’ package.

### 2.4 Creating and verifying the DRL signature

An algorithm was developed to measure disulfidptosis patterns in each individual with CRC, providing a prognosis feature. To prevent overfitting and create the best prognosis feature called the DRL score’, we utilized lasso regression analysis.


$$\begin{array}{l} \text{DRL score}=\left[\text{SNHG}17\text{ expression}\times \left(0.262\right)\right]+\left[\text{ALMS}1-\text{IT}1\text{ expression}\times \left(0.244\right)\right]\\ +\left[\text{AC}087481.3\text{ expression}\times \left(0.038\right)\right]+\left[\text{AL}138724.1\text{ expression}\times \left(0.106\right)\right]\;\\ +\;\left[\text{AC}069281.2\text{ expression}\times \left(0.149\right)\right]+\left[\text{NCK}1-\text{DT expression}\times \left(0.098\right)\right]\\ +\;\left[\text{AC}024560.3\text{ expression}\times \left(0.078\right)\right]+\left[\text{SNHG}26\text{ expression}\times \left(0.184\right)\right]\\ +\;\left[\text{AP}001505.1\text{ expression}\times \left(0.137\right)\right]+\left[\text{HOXC}-\text{AS}2\text{ expression}\times \left(0.034\right)\right]\\ +\;\left[\text{AC}018653.3\text{ expression}\times \left(0.117\right)\right]+\left[\text{SNHG}16\text{ expression}\times \left(-0.350\right)\right] \end{array}$$

Using the ‘caret’ package, the TCGA database was split into training and testing sets. DRL’s signature was validated using Kaplan-Meier survival analysis using the “survival” and “survminer” packages. The prognosis model’s sensitivity and accuracy were assessed using the ‘survivalROC’ package. Using the R packages “limma” and “scatterplot3d”, all CRC samples were subjected to principal component analysis (PCA). A comparison of DRL’s signature with other lncRNA-based CRC prognostic features was conducted using the “limma,” “Survival,” “survminer,” and “timeROC” packages.

### 2.5 Evaluation of the DRL signature’s clinical usefulness and independent prognostic analysis

The independence of the DRL score from other clinical features was evaluated through univariate and multivariate Cox regression analyses. The predictive capability of DRL scores in different subgroups was evaluated through a stratified analysis. “limma” and “ggpubr” packages were used to evaluate the correlation between DRL score and immune score. Associations among DRL score, immune score, Microsatellite Instability (MSI), age, sex, and TNM staging were examined using the ‘limma’ and ‘ggpubr’ packages. Additionally, a nomogram was developed to forecast the overall survival (OS) of colorectal cancer (CRC) patients at 1 year, 3 years, and 5 years by integrating the DRL score with various clinical characteristics. The nomogram’s accuracy was evaluated using calibration plots from the ‘regplot,’ ‘survival,’ and ‘rms’ packages.

### 2.6 Analysis of immune microenvironment features in CRC subcategories based on DRL scores

To evaluate variances in the immune microenvironment features among various DRL score subcategories in CRC, we employed the ssGSEA and ESTIMATE algorithms. The evaluation of immune cell infiltration and function in CRC patients was performed for the ‘GSVA’ package through single-sample gene set enrichment analysis (ssGSEA). In medical research, ssGSEA, a commonly employed enrichment algorithm, measures the proportionate prevalence of every cell infiltration within the CRC tumor microenvironment (TME).

### 2.7 Cell culture

The Chinese Academy of Sciences’ Cell Bank (in Shanghai, China) provided the human NCM460, HCT116, DLD1, and RKO cells for culture. In DMEM with 10% FBS, 1% antibiotics, and 37°C with 5% CO2, NCM460, HCT116, DLD1, and RKO cells were grown.

### 2.8 Real-time PCR with quantification (qRT-PCR)

Trizol (Sigma) was used to extract total RNA from cell lines. 1 microgram of total RNA was reverse transcribed into complementary DNA using the Rever TraAce qPCR RT kit from Toyota after being quantified with a Nanodrop 1000 spectrophotometer (Thermo Scientific). Using the StepOne real-time PCR machine from Applied Biosystems, quantitative PCR was carried out using the SYBR Green real-time PCR Master Mix from Toyobo. As an internal control, GAPDH was utilized. In **Supplementary Table S1**, primer sequences for the pertinent genes are listed.

### 2.9 Statistical analysis

All statistical analyses were performed using the R software program, version 4.2.1. Using one-way ANOVA or a Student’s t-test, statistical differences between groups were computed. Unless otherwise noted, P< 0.05 was assumed to be statistically significant.

## 3 Result

### 3.1 Expression and mutation landscape of DRGs

**Figure 1:**
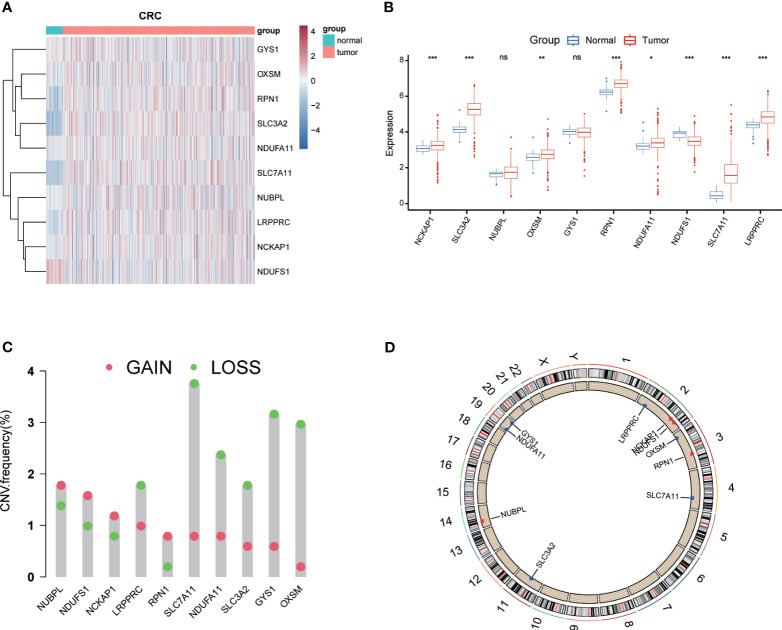
Expression levels and genetic variations of Disulfidptosis-Related Genes (DRGs). Heatmap **(A)** and boxplot **(B)** showing the expression levels of DRGs in colorectal cancer and normal tissues. Mutation frequency of DRGs in colorectal cancer patients **(C)**. **(D)** Chromosomal positions of DRGs. * p< 0.05; ** p< 0.01; *** p< 0.001; ns p> 0.05.

To investigate the potential role of DRGs in CRC progression, 10 DRGs (GYS1, NDUFS1, NDUFA11, OXSM, LRPPRC, NUBPL, NCKAP1, RPN1, SLC3A2, and SLC7A11) were obtained from Liu’s study (10). Among them, 8 DRGs (NDUFS1, NDUFA11, OXSM, LRPPRC, NCKAP1, RPN1, SLC3A2, and SLC7A11) displayed a marked differential expression pattern between CRC and normal tissues (P<0.05) (**Figure 1A**). Notably, NDUFA11, OXSM, LRPPRC, NCKAP1, RPN1, SLC3A2, and SLC7A11 were upregulated in cancer tissues, while NDUFS1 showed the opposite trend (**Figure 1B**). Following that, the analysis of CNV data using Gistic2.0 identified chromosomal segments with significant amplifications and deletions, comparing CNVs across chromosomal arms. The visualization of gene chromosomal positions using “RCircos” in R revealed that copy number deletions were the predominant mutations in DRGs (**Figure 1C**). The most significant alterations were observed in SLC7A11, OXSM, and GYS1, in contrast to NUBPL, NDUFS1 and NCKAP1 that predominantly exhibited copy number amplifications. Since CNV is a significant component of structural variation (SV) in the genome, the chromosomal positions of CNV mutations in DRGs were represented in the circular plot (**Figure 1D**).

### 3.2 Identification of prognostic-related DRLs in CRC

**Figure 2:**
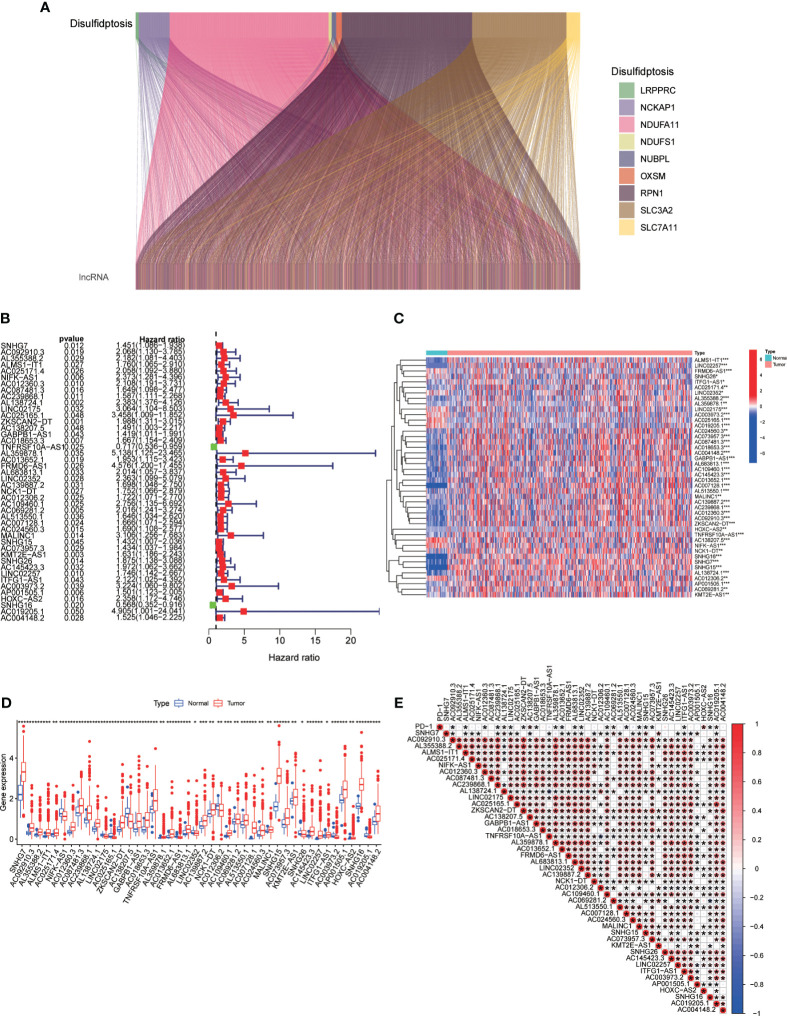
**(A)** Sankey diagram depicting the relationship between double sulfur death-related genes and co-expressed lncRNAs. **(B)** Prognosis-relevant DRLs identified through single-factor Cox regression analysis. **(C, D)** Heatmap **(C)** and boxplot **(D)** displaying expression levels of DRLs. **(E)** Correlation between prognosis-related DRLs and PD-L1 expression. *p< 0.05, **p< 0.01, ***p< 0.001.

The association between genes related to disulfidptosis and lncRNAs was evaluated using a Pearson correlation analysis and a Sankey diagram was used for visualization (**Figure 2A**). To validate the prognostic potential of DRLs, we determined 44 prognostic-related DRLs through univariate Cox regression analysis. Among them, 42 lncRNAs were identified as high-risk lncRNAs (HR > 1), while the other two were identified as low-risk lncRNAs (hazard ratio (HR) < 1) (**Figure 2B**). Besides, we assessed the expression levels of these prognostic-related DRLs and found that all 44 DRLs showed a differential expression pattern between CRC tissues and control ones (**Figure 2C**). Specifically, 34 DRLs were upregulated, while 10 DRLs were downregulated in CRC samples (**Figure 2D**). Furthermore, we utilized the “limma” and “corrplot” packages to explore the correlation between the expression of the immune checkpoint gene PD-L1 and DRLs. Our results demonstrate a positive correlation between AC138207.5, AC069281.2, HOXC−AS2, AC019205.1, and PD-1, while AC092910.3, ALMS1−IT1, AC012360.3, AC239868.1, LINC02175, ZKSCAN2−DT, GABPB1−AS1, L359878.1, AC013652.1, AL683813.1, LINC02352, AC109460.1, AC007128.1, MALINC1, SNHG15, AC073957.3, ITFG1−AS1, and SNHG16 show a negative correlation with PD-1 (**Figure 2E**). All these results demonstrate strong correlations, thereby suggesting a significant potential role for DRLs in predicting prognosis in colorectal cancer.

### 3.3 CRC molecular subgroups based on DRLs

**Figure 3:**
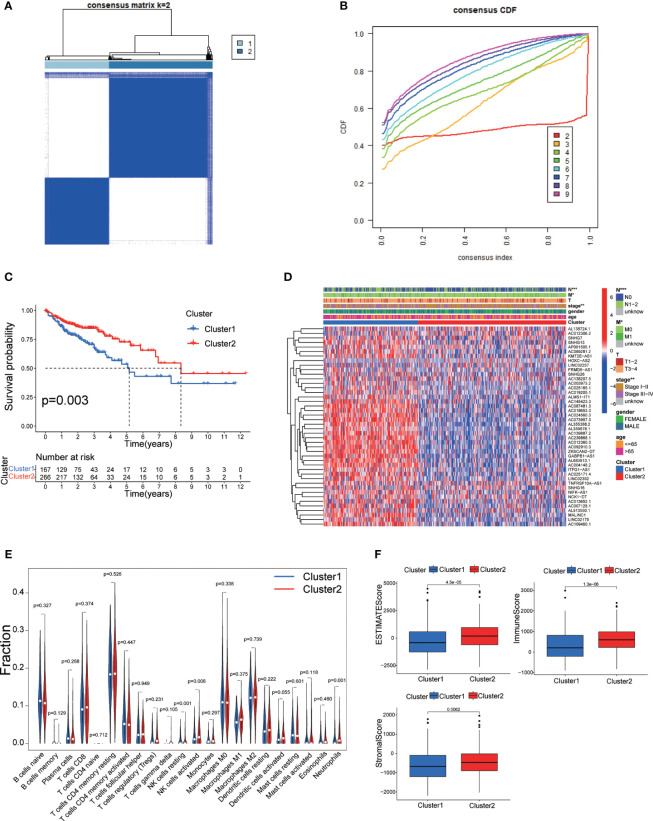
**(A)** Consensus clustering matrix for k = 2. **(B)** Cumulative distribution function curve for K = 2–10. **(C)** Survival analysis of two molecular subtypes. **(D)** Heatmap depicting differential expression of prognosis-related DRLs among different subtypes. **(E)** Immune cell infiltration landscape in the two subtypes. **(F)** ESTIMATE score, immune score, and stromal score comparison between the two subtypes. DRLs, disulfidptosis-related. * p< 0.05; ** p< 0.01; *** p< 0.001; ns p> 0.05.

To further investigate the expression profile of DRLs in CRC, we performed consensus clustering analysis and divided CRC patients into two subgroups (Cluster 1 and Cluster 2) based on the expression of fore-mentioned DRLs. Survival rates indicated that Cluster 2 patients had a more significant survival advantage than those in Cluster 1 (**Figure 3C**). The heatmap data demonstrated a differential expression pattern of prognostic-related DRLs in the two clusters, and the majority of lncRNAs showed higher expression in Cluster 1 than in Cluster 2 (**Figure 3D**). Cluster-1 was characterized by increased expressions of AC145423.3, AC018653.3, AC087481.3, AC024560.3, AC073957.3, AL355388.2, AL359878.1, AC139887.2, AC239868.1, AC012360.3, AC092910.3, ZKSCAN2−DT, GABPB1−AS1, AL683813.1, and AC004148.2. In addition, we analyzed the immune cell infiltration landscape in different clusters, revealing that various immune cell types were significantly enriched in Cluster 2 (**Figure 3E**). Moreover, the ESTIMATE score, immune score, and stromal score were significantly higher in Cluster 2 than those of Cluster 1 (**Figure 3F**), indicating that cluster analysis based on DRLs exhibits remarkable effectiveness.

### 3.4 Validation of DRLs’ prognostic value in CRC

**Figure 4:**
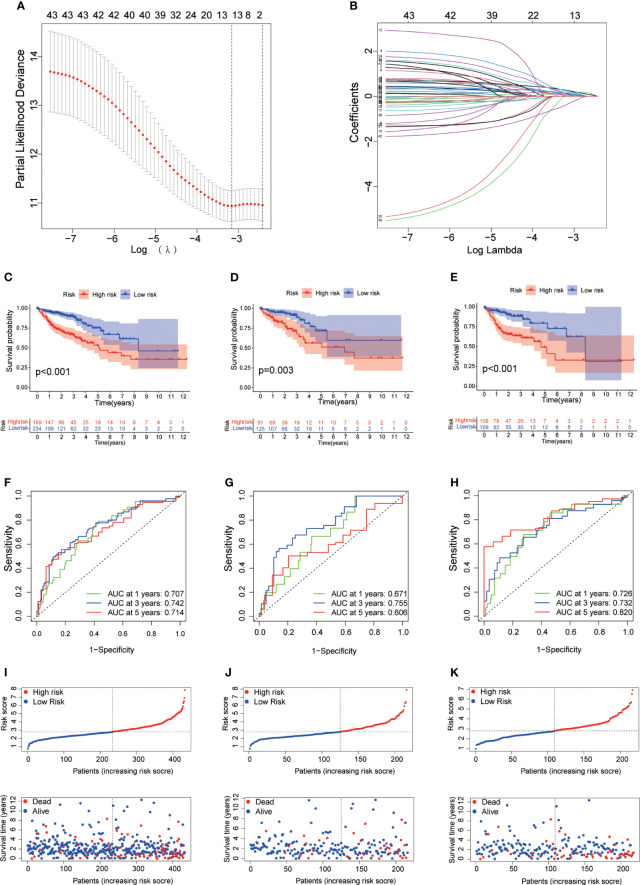
**(A)** Determination of Optimal λ Value. **(B)** LASSO Coefficient Profiles of DRLs. **(C–E)** Survival Analysis Based on DRL-Score: Training **(C)**, Validation **(D)**, and Entire Cohort **(E)**. **(F–H)** ROC Curve for Overall Survival Prediction: Training **(F)**, Validation **(G)**, and Entire Cohort **(H)**. **(I–K)** Distribution of DRL-Score, Patient Survival Status, and Survival Time: Training **(I)**, Validation **(J)**, and Entire Cohort **(K)**. DRLs, disulfidptosis-related lncRNAs.

**Figure 5:**
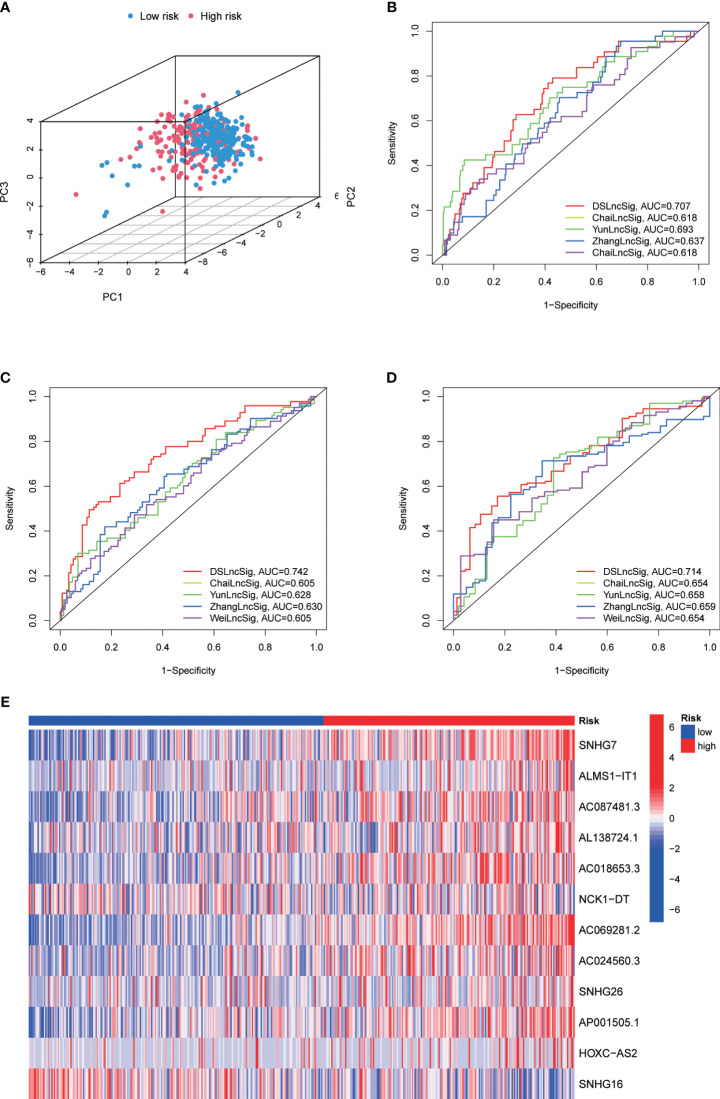
**(A)** Principal Component Analysis for 1-Year **(B)**, 3-Year **(C)**, and 5-Year **(D)** Overall Survival in the TARGET Cohort. **(E)** Heatmap Displaying Expression of Twelve Disulfidptosis-Related lncRNAs.

To precisely predict the prognosis of CRC patients, we constructed a prognostic model for DRLs using 12 key DRLs determined by univariate Cox and LASSO regression analyses (**Figures 4A, B**). These 12 DRLs are closely associated with GYS1, NDUFS1, NDUFA11, OXSM, LRPPRC, NUBPL, NCKAP1, RPN1, SLC3A2, and SLC7A11 (**Supplementary Figure 1A**), which are intimately correlated to a poorer prognosis of CRC except for SNHG16 (**Supplementary Figure 1B**). Consequently, we categorized individuals into low-risk and high-risk categories depending on the median DRL score. The Kaplan-Meier analysis demonstrated that individuals in the high-risk category experienced considerably poorer overall survival (OS) compared to those in the low-risk category, as observed in both the training and validation sets (**Figures C–E**). Additionally, ROC analysis showed that the DRL score efficiently predicted the overall survival tendency of CRC patients in the training set, validation set, and entire cohort (**Figures 4F–H**). Furthermore, PCA analysis demonstrated distinct clustering of patients based on the DRL score (**Figure 5A**). The AUC values of DRL-associated features for 1-year, 3-year, and 5-year OS were significantly higher than those of other clinical features, indicating a high accuracy of the DRL-based prognostic model (**Figures 5B–D**). Heatmap analysis displayed a remarkable difference in the expression of 12 key DRLs between the CRC patients in the low-risk group and those in the high-risk group (**Figure 5F**).

### 3.5 Clinical correlation analysis and stratified analysis using a DRL-based pronostic model

**Figure 6:**
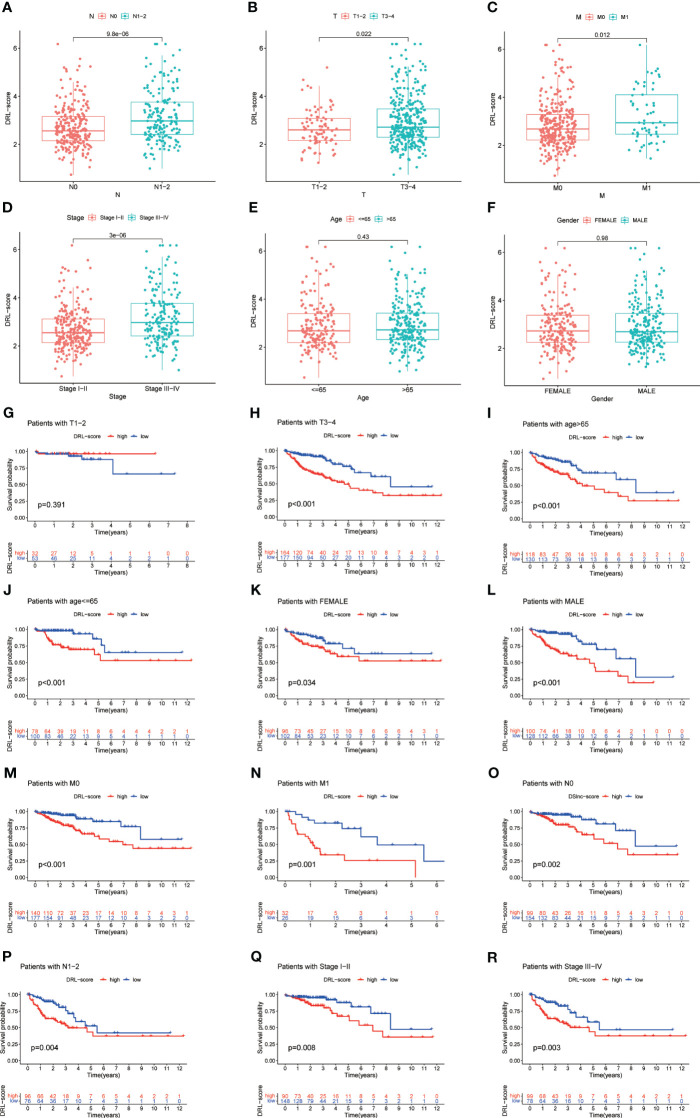
Correlation Between DRL Score and Clinical Factors **(A-F)**. Stratified Survival Analysis for Various Clinical Subgroups **(G-R)**. T, tumor size; N, lymph node involvement; M, metastasis.

The analysis of clinical correlation showed that patients with colorectal cancer who had a high DRL score exhibited larger tumors, more lymph node involvement, a more advanced tumor stage, and a wider distribution of primary lesions. Nevertheless, there were no notable disparities in DRL scores among patients of varying ages and genders (**Figures 6A–F**).The predictive capability of the DRL score in different clinical subgroups was further confirmed through stratified survival analysis, as higher DRL scores showed a strong association with poorer overall survival in the majority of subgroups (**Figures 6G–R**).

**Figure 7:**
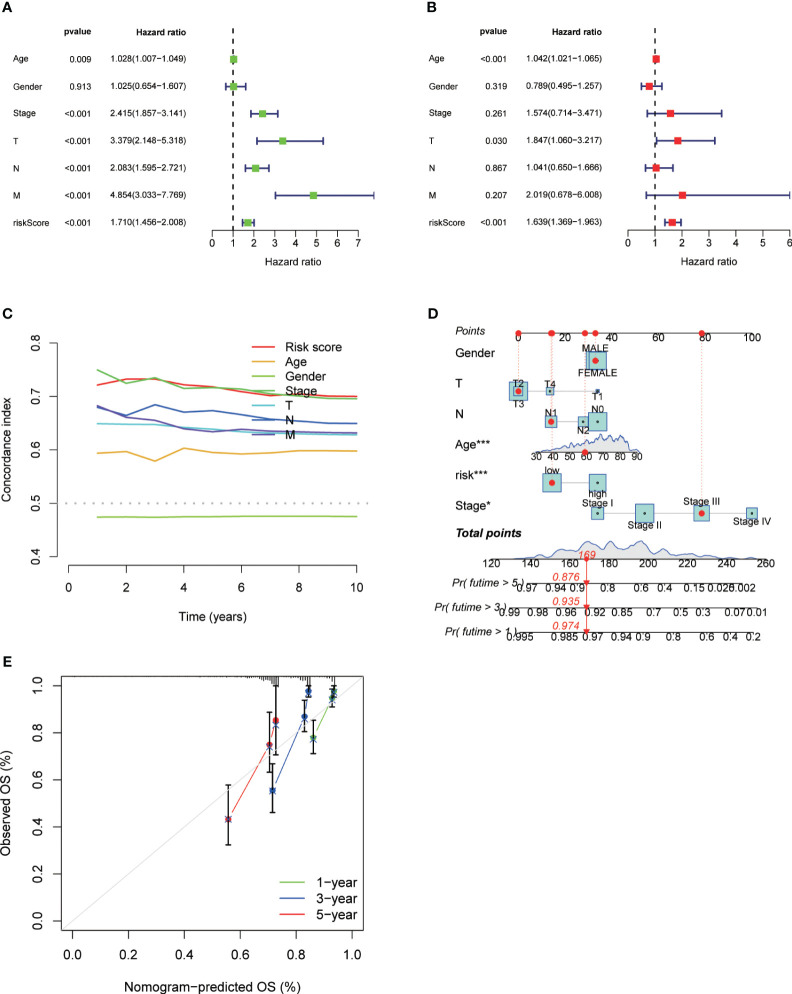
Univariate Analysis **(A)**, Multivariate Analysis **(B)**, and Concordance Index **(C)** for Different Clinical Parameters and DRL Scores. Construction of Forest Plots Utilizing Different Clinical Parameters and DRL Scores **(D)**. Calibration Plots of Forest Plots for Overall Survival Prediction **(E)**. * p< 0.05; ** p< 0.01; *** p< 0.001; ns p> 0.05.

We performed univariate and multivariate Cox regression analysis and Concordance Index analysis to confirm the autonomous predictive ability of the DRL score. This analysis revealed that both the DRL score and stage could act as autonomous prognostic markers for patients with CRC (**Figures 7A–C**).By utilizing calibration curves, the constructed column chart successfully forecasted the prognosis of CRC patients at various time intervals (**Figures 7D, E**).

### 3.6 Characteristics of the immune microenvironment in various DRL score subgroups

**Figure 8:**
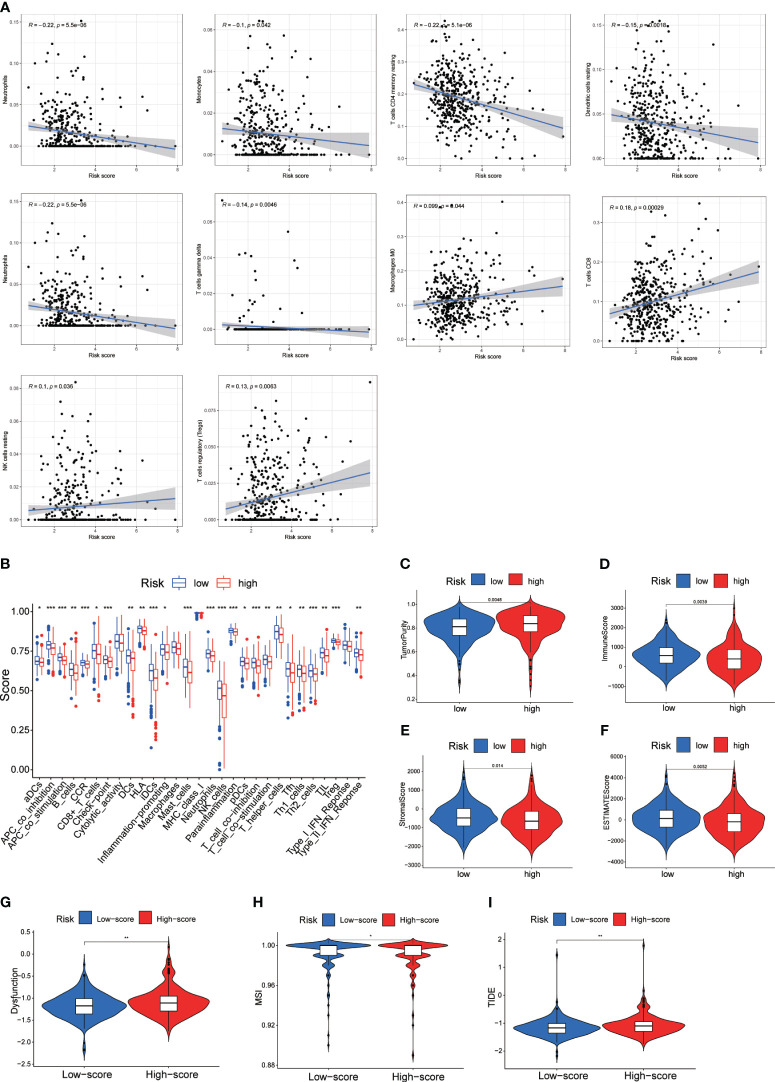
**(A)** Correlation Analysis Between DRL Score and Immune Infiltrating Cells. **(B)** Single-Sample Gene Set Enrichment Analysis (ssGSEA) of Immune Infiltrating Cells in Different DRL Score Subgroups. **(C-F)** Immune Scores Among Different DRL Score Subgroups. **(G-I)** Immune Therapy Scores Across Different DRL Score Subgroups. * p< 0.05; ** p< 0.01; *** p< 0.001.

We conducted a study on the role of DRL scores in predicting the immune microenvironment landscape in CRC using the CIBERSORT algorithm. The results of Pearson correlation analysis revealed a significant negative correlation between DRL scores and various immune cell types. Specifically, DRL scores were negatively correlated with eosinophils, neutrophils, monocytes, CD4 memory resting T cells, resting dendritic cells, and T cells gamma delta. In contrast, DRL scores showed a positive correlation with CD8 T cells, regulatory T cells (Tregs), resting NK cells, and macrophages M0 (**Figure 8A**). This implies that individuals with elevated DRL scores might encounter a reduction in the abundance of various immune cell types, potentially resulting in the suppression or modulation of immune system functions throughout the anti-tumor process.

Further analysis of tumor infiltration indicated that the high DRL score group exhibited lower levels of various immune cell types compared to the low DRL score group (**Figures 8B**). Additionally, the high DRL score group showed lower immune, stromal, and ESTIMATE scores, along with higher tumor purity (**Figures 8C–F**). Consequently, we speculate that patients with high DRL scores may have a poorer prognosis due to restricted immune cell infiltration. Moreover, the high DRL score group demonstrated significantly elevated scores for functional impairment, MSI, and TIDE (**Figures 8G–I**). The high MSI status may render tumor cells more susceptible to recognition and attack by the immune system. The increased functional impairment score suggests that these patients’ immune systems may experience inhibition or modulation, resulting in reduced immune cell activity and challenges in effectively combating tumor cells. The elevated TIDE score may indicate stronger immune evasion features in these patients’ tumors, posing greater challenges for immune therapy. Overall, the negative correlations imply lower immune system activity in certain aspects for patients with high DRL scores, potentially enabling tumors to more effectively evade immune surveillance, exhibit more invasive biological behavior, and present greater challenges for treatment.

### 3.7 *In vitro* validation of the DRL-based prognosis prediction model in CRC

**Figure 9:**
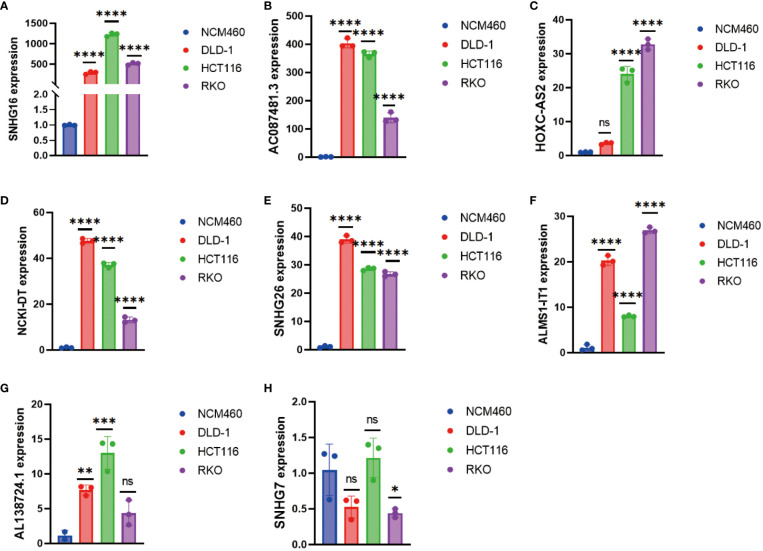
Detection of **(A)** SNHG16, **(B)** AC087481.3, **(C)** HOXC-AS2, **(D)** NCK1-DT, **(E)** SNHG26, **(F)** ALMS1-IT1, **(G)** AL138724.1, and **(H)** SNHG7 expression in colorectal cancer cell lines by RT-qPCR. * p< 0.05; ** p< 0.01; *** p< 0.001, **** p< 0.0001.

As mentioned above, we had performed various analyses to demonstrate that our DRL-based prediction is highly consistent with clinical data. To further validate the findings, we conducted RT-qPCR analysis on CRC cell lines (DLD-1, HCT116, and RKO) and the normal cell line NCM460 to avoid background noise caused by hetereogenous cell types from *in vivo* tumor tissues. The results confirmed overall significant up-regulated levels of SNHG16, HOXC-AS2, SNHG26, AC087481.3, AL138724.1, NCK1-DT, and ALMS1-IT1 in CRC cell lines compared to those of normal cell lines (**Figure 9**). In summary, both bioinformatic analyses and *in vitro* experiments support our proposed DRL-based model as an effective strategy for CRC prognosis prediction, which may shed light on precise medicine in CRC treatment by using certain DRL scores as typical standards for strategic decision-making.

## 4 Discussion

Early screening advancements have positively impacted colorectal cancer survival rates, yet it remains a major global cause of cancer-related deaths (22). In the realm of treatment progress, immunotherapy, specifically immune checkpoint blockade (ICB), has emerged as a promising avenue for certain colorectal cancer patients (23). Notably, Keytruda (pembrolizumab), an anti-PD-1 monoclonal antibody, has received FDA approval for the first-line treatment of unresectable or metastatic microsatellite instability-high (MSI-H) or mismatch repair-deficient (dMMR) colorectal cancer patients, offering newfound hope for those resistant to traditional chemotherapy (24). While significant progress has been achieved, a substantial proportion of colorectal cancer patients lack the specific alterations targeted by immune checkpoint blockade (ICB). This emphasizes the urgent need to uncover the cellular and molecular factors contributing to immunotherapy resistance (25–27).. Hence, it is crucial to discover new indicators and treatment objectives to enhance the results of individuals with CRC.

Biological development and internal environmental balance are both maintained by cell death, a physiological process (28, 29). Targeting cell death-related pathways to eliminate cancer cells is a major direction in cancer treatment (28, 30, 31). Recently, a research team discovered and identified a novel type of cell death called disulfidptosis. The discovery of disulfidptosis (DSD) as a distinct mode of cell death introduces a novel perspective on cancer progression and treatment strategies (32). The connection between DSD and the actin cytoskeleton underscores its potential to influence key cellular processes, potentially paving the way for therapeutic interventions (14, 32, 32). Understanding how DSD influences tumor initiation, progression, metastasis, and treatment resistance is crucial for developing effective strategies. While current therapies often aim to induce apoptosis, resistance to apoptosis leads to treatment failure and disease recurrence. This underscores the importance of exploring DSD as a potential diagnostic and therapeutic avenue (33).

lncRNAs have emerged as integral players in various biological processes, offering potential as diagnostic, prognostic, and therapeutic tools (34, 35). Their multifaceted roles include regulating gene expression through interactions with proteins, RNA, and DNA, thereby impacting critical cellular functions (34). Notably, lncRNAs have been implicated in CRC development and progression, rendering them attractive candidates for early diagnosis and treatment (36). As a result, lncRNAs represent a novel avenue for advancing CRC management.

Currently, bioinformatics analysis has been widely applied in the detection, diagnosis, treatment, and drug screening of tumors, providing essential tools and methods for more effective medical practices (37–40). Our study investigated the potential role of DRLs in CRC and aimed to develop a prognostic model for predicting patient outcomes and guiding clinical decisions. This study is the first comprehensive exploration of the prognostic significance of DRLs in CRC. We initially investigated the expression and mutation profiles of 10 DRGs in CRC tissues, identifying 8 DRGs with significant differential expression between CRC and normal tissues (**Figure 1**). Subsequently, using univariate Cox regression analysis, we identified 44 prognostic-related DRLs, of which 42 were classified as high-risk and 2 as low-risk for CRC patients. All 44 DRLs showed differential expression between CRC samples and control tissues (**Figure 2**). To study the molecular subgroups of CRC based on DRL expression, we conducted consensus clustering, resulting in the division of patients into two subgroups (Cluster 1 and Cluster 2). Cluster 2 patients showed a significant survival advantage compared to Cluster 1 patients. Immune cell infiltration analysis revealed various immune cell types significantly enriched in Cluster 2 (**Figure 3**). All these data implicate a potential immune-related mechanism underlying different survival outcomes, in which DRL may take an unignorable part.

To construct a relevant prognostic model, we used 12 key DRLs (SNHG17, ALMS1-IT1, AC087481.3, AL138724.1, AC069281.2, NCK1-DT, AC024560.3, SNHG26, AP001505.1, HOXC-AS2, SNHG16) identified through univariate Cox and LASSO regression analysis (**Figure 4**). The DRL score model effectively predicted the survival of CRC patients in both the training and validation sets (**Figure 5**). Moreover, the DRL score demonstrated higher accuracy compared to other clinical features, highlighting its potential clinical utility as a prognostic tool (**Figures 6**, **7**). Notably, some DRLs, such as SNHG17, SNHG26, and SNHG16, have been previously linked to colorectal tumorigenesis and metastasis (41–43), while NCK1-DT and HOXC-AS2 expression were associated with immune cell infiltration heterogeneity (44), suggesting that DRLs may aid in predicting survival outcomes and evaluating cancer immune regulation. Additionally, our study revealed, for the first time, the impact of AL138724.1, AC069281.2, AC024560.3, and AP001505.1 on prognosis in CRC (**Supplementary Figure 1**), which may open a new window for further investigation as to other cancer types.

As well-known, TME plays a crucial role in tumor initiation and development and is a critical determinant of prognosis and treatment response in CRC patients. Particularly, tumor-infiltrating CD8+ T cells are key players in effective anti-tumor responses. Our study found the DRL score was associated with specific immune cell types, such as B cells, CD8+ T cells, and dendritic cells, among others. The low DRL score group exhibited higher levels of immune cell infiltration, lower immune scores, stromal scores, and ESTIMATE scores, and higher tumor purity (**Figure 9**). This suggests that the DRL score might influence the immune microenvironment and impact tumor progression. To validate this finding, we performed RT-qPCR analysis on CRC cell lines and normal cell lines, confirming the significant upregulation of DRLs in CRC cell lines compared to normal cell lines (**Figure 8**). Therefore, we proposed the first DRL-based prognosis prediction model for CRC with higher accuracy than current biomarkers, which also offered an in-depth understanding of the clinically applicable role of lncRNAs in cancer treatment.

## 5 Conclusion

In conclusion, this study identified key DRLs associated with CRC prognosis. The developed DRL score model showed promising potential as a prognostic tool to predict individual patient outcomes and guide clinical decisions. Additionally, the study shed light on the immune microenvironment characteristics associated with DRLs, providing insights into potential immunotherapy targets for CRC treatment.

## Data availability statement

The datasets presented in this study can be found in online repositories. The names of the repository/repositories and accession number(s) can be found in the article/**Supplementary Material**.

## Author contributions

CD: Data curation, Visualization, Writing – original draft, Writing – review & editing, Validation. YG: Supervision, Writing – original draft. PW: Formal Analysis, Validation, Writing – original draft. SY: Investigation, Methodology, Visualization, Writing – original draft. XG: Supervision, Writing – original draft, Writing – review & editing, Funding acquisition.

## Supplementary material

Supplementary Figure 1
**(A)** Correlation heatmap of the Disulfidptosis-Related Gene Set and Disulfidptosis-Related lncRNAs. DRGs, disulfidptosis-related genes; DRLs, disulfidptosis-related lncRNAs. **(B)** Survival analysis of the 12 Disulfidptosis-Related lncRNAs in Colorectal Cancer.

## References

[1] Siegel RL Miller KD Wagle NS Jemal A. Cancer statistics, 2023. CA Cancer J Clin (2023) 73(1):17–48. doi: 10.3322/caac.21763 36633525

[2] Siegel RL Miller KD Fuchs HE Jemal A. Cancer statistics, 2022. CA Cancer J Clin (2022) 72(1):7–33. doi: 10.3322/caac.21708 35020204

[3] Siegel RL Miller KD Fuchs HE Jemal A. Cancer statistics, 2021. CA Cancer J Clin (2021) 71(1):7–33. doi: 10.3322/caac.21654 33433946

[4] Vasaikar S Huang C Wang X Petyuk VA Savage SR Wen B. Proteogenomic analysis of human colon cancer reveals new therapeutic opportunities. Cell (2019) 177(4):1035–49.e19. doi: 10.1016/j.cell.2019.03.030 31031003 PMC6768830

[5] Lech G Słotwiński R Słodkowski M Krasnodębski IW. Colorectal cancer tumour markers and biomarkers: Recent therapeutic advances. World J Gastroenterol (2016) 22(5):1745–55. doi: 10.3748/wjg.v22.i5.1745 PMC4724606 26855534

[6] Heinemann V von Weikersthal LF Decker T Kiani A Vehling-Kaiser U Al-Batran SE. FOLFIRI plus cetuximab versus FOLFIRI plus bevacizumab as first-line treatment for patients with metastatic colorectal cancer (FIRE-3): a randomised, open-label, phase 3 trial. Lancet Oncol (2014) 15(10):1065–75. doi: 10.1016/S1470-2045(14)70330-4 25088940

[7] Van Cutsem E Cervantes A Adam R Sobrero A Van Krieken JH Aderka D. ESMO consensus guidelines for the management of patients with metastatic colorectal cancer. Ann Oncol (2016) 27(8):1386–422. doi: 10.1093/annonc/mdw235 27380959

[8] Liu X Nie L Zhang Y Yan Y Wang C Colic M. Actin cytoskeleton vulnerability to disulfide stress mediates disulfidptosis. Nat Cell Biol (2023) 25(3):404–14. doi: 10.1038/s41556-023-01091-2 PMC10027392 36747082

[9] Zheng P Zhou C Ding Y Duan S. Disulfidptosis: a new target for metabolic cancer therapy. J Exp Clin Cancer Res (2023) 42(1):103. doi: 10.1186/s13046-023-02675-4 37101248 PMC10134647

[10] Liu X Olszewski K Zhang Y Lim EW Shi J Zhang X. Cystine transporter regulation of pentose phosphate pathway dependency and disulfide stress exposes a targetable metabolic vulnerability in cancer. Nat Cell Biol (2020) 22(4):476–86. doi: 10.1038/s41556-020-0496-x PMC7194135 32231310

[11] Chen SL Huang QS Huang YH Yang X Yang MM He YF. GYS1 induces glycogen accumulation and promotes tumor progression *via* the NF-κB pathway in Clear Cell Renal Carcinoma. Theranostics (2020) 10(20):9186–99. doi: 10.7150/thno.46825 PMC7415807 32802186

[12] Wang Y Wu N Sun D Sun H Tong D Liu D. NUBPL, a novel metastasis-related gene, promotes colorectal carcinoma cell motility by inducing epithelial-mesenchymal transition. Cancer Sci (2017) 108(6):1169–76. doi: 10.1111/cas.13243 PMC5480060 28346728

[13] Gu J Wu M Guo R Yan K Lei J Gao N. The architecture of the mammalian respirasome. Nature (2016) 537(7622):639–43. doi: 10.1038/nature19359 27654917

[14] Lu W Zhang Y McDonald DO Jing H Carroll B Robertson N. Dual proteolytic pathways govern glycolysis and immune competence. Cell (2014) 159(7):1578–90. doi: 10.1016/j.cell.2014.12.001 PMC4297473 25525876

[15] Cui J Wang L Ren X Zhang Y Zhang H. LRPPRC: A multifunctional protein involved in energy metabolism and human disease. Front Physiol (2019) 10:595. doi: 10.3389/fphys.2019.00595 31178748 PMC6543908

[16] Gao T Qian S Shen S Zhang X Liu J Jia W. Reduction of mitochondrial 3-oxoacyl-ACP synthase (OXSM) by hyperglycemia is associated with deficiency of α-lipoic acid synthetic pathway in kidney of diabetic mice. Biochem Biophys Res Commun (2019) 512(1):106–11. doi: 10.1016/j.bbrc.2019.02.155 30871779

[17] Machesky LM. Deadly actin collapse by disulfidptosis. Nat Cell Biol (2023) 25(3):375–6. doi: 10.1038/s41556-023-01100-4 36918690

[18] Tang X Qiao X Chen C Liu Y Zhu J Liu J. Regulation mechanism of long noncoding RNAs in colon cancer development and progression. Yonsei Med J (2019) 60(4):319–25. doi: 10.3349/ymj.2019.60.4.319 PMC6433576 30900417

[19] Yao ZT Yang YM Sun MM He Y Liao L Chen KS. New insights into the interplay between long non-coding RNAs and RNA-binding proteins in cancer. Cancer Commun (Lond) (2022) 42(2):117–40. doi: 10.1002/cac2.12254 PMC8822594 35019235

[20] Ma Z Peng P Zhou J Hui B Ji H Wang J. Long non-coding RNA SH3PXD2A-AS1 promotes cell progression partly through epigenetic silencing P57 and KLF2 in colorectal cancer. Cell Physiol Biochem (2018) 46(6):2197–214. doi: 10.1159/000489589 29734178

[21] Zong S Dai W Guo X Wang K. LncRNA-SNHG1 promotes macrophage M2-like polarization and contributes to breast cancer growth and metastasis. Aging (Albany NY) (2021) 13(19):23169–81. doi: 10.18632/aging.203609 PMC8544328 34618681

[22] Fitzmaurice C Abate D Abbasi N Abbastabar H Abd-Allah F Abdel-Rahman O. Global, regional, and national cancer incidence, mortality, years of life lost, years lived with disability, and disability-adjusted life-years for 29 cancer groups, 1990 to 2017: A systematic analysis for the global burden of disease study. JAMA Oncol (2019) 5(12):1749–68. doi: 10.1001/jamaoncol.2019.2996 PMC6777271 31560378

[23] André T Shiu KK Kim TW Jensen BV Jensen LH Punt C. Pembrolizumab in microsatellite-instability–high advanced colorectal cancer. N Engl J Med (2020) 383:2207–18. doi: 10.1056/NEJMoa2017699 33264544

[24] Le DT Durham JN Smith KN Wang H Bartlett BR Aulakh LK. Mismatch repair deficiency predicts response of solid tumors to PD-1 blockade. Science (2017) 357:409–13. doi: 10.1126/science.aan6733 PMC5576142 28596308

[25] Bray F Ferlay J Soerjomataram I Siegel RL Torre LA Jemal A. Global cancer statistics 2018: GLOBOCAN estimates of incidence and mortality worldwide for 36 cancers in 185 countries. CA Cancer J Clin (2018) 68(6):394–424. doi: 10.3322/caac.21492 30207593

[26] Chen W Zheng R Baade PD Zhang S Zeng H Bray F. Cancer statistics in China, 2015. CA Cancer J Clin (2016) 66(2):115–32. doi: 10.3322/caac.21338 26808342

[27] Arnold M Sierra MS Laversanne M Soerjomataram I Jemal A Bray F. Global patterns and trends in colorectal cancer incidence and mortality. Gut (2017) 66(4):683–91. doi: 10.1136/gutjnl-2015-310912 26818619

[28] Galluzzi L Vitale I Aaronson SA Abrams JM Adam D Agostinis P. Molecular mechanisms of cell death: recommendations of the Nomenclature Committee on Cell Death 2018. Cell Death Differ (2018) 25(3):486–541. doi: 10.1038/s41418-017-0012-4 29362479 PMC5864239

[29] Gaschler MM Andia AA Liu H Csuka JM Hurlocker B Vaiana CA. FINO(2) initiates ferroptosis through GPX4 inactivation and iron oxidation. Nat Chem Biol (2018) 14(5):507–15. doi: 10.1038/s41589-018-0031-6 PMC5899674 29610484

[30] Green DR Llambi F. Cell death signaling. Cold Spring Harb Perspect Biol (2015) 7(12):2–4. doi: 10.1101/cshperspect.a006080 PMC4665079 26626938

[31] Fuchs Y Steller H. Programmed cell death in animal development and disease. Cell (2011) 147(4):742–58. doi: 10.1016/j.cell.2011.10.033 PMC4511103 22078876

[32] Liu DS Duong CP Haupt S Montgomery KG House CM Azar WJ. Inhibiting the system x(C)(-)/glutathione axis selectively targets cancers with mutant-p53 accumulation. Nat Commun (2017) 8:14844. doi: 10.1038/ncomms14844 28348409 PMC5379068

[33] He Y Xu Y Zhang C Gao X Dykema KJ Martin KR. Identification of a lysosomal pathway that modulates glucocorticoid signaling and the inflammatory response. Sci Signal (2011) 4(180):ra44. doi: 10.1126/scisignal.2001450 21730326 PMC3684214

[34] Chen S Shen X. Long noncoding RNAs: functions and mechanisms in colon cancer. Mol Cancer (2020) 19(1):167. doi: 10.1186/s12943-020-01287-2 33246471 PMC7697375

[35] Mercer TR Dinger ME Mattick JS. Long non-coding RNAs: insights into functions. Nat Rev Genet (2009) 10(3):155–9. doi: 10.1038/nrg2521 19188922

[36] Gupta RA Shah N Wang KC Kim J Horlings HM Wong DJ. Long non-coding RNA HOTAIR reprograms chromatin state to promote cancer metastasis. Nature (2010) 464(7291):1071–6. doi: 10.1038/nature08975 PMC3049919 20393566

[37] Su K Wang F Li X Chi H Zhang J He K. Effect of external beam radiation therapy versus transcatheter arterial chemoembolization for non-diffuse hepatocellular carcinoma (≥ 5 cm): a multicenter experience over a ten-year period. Front Immunol (2023) 14:1265959. doi: 10.3389/fimmu.2023.1265959 37818373 PMC10560878

[38] Li H Wu Z Chen J Su K Guo L Xu K. External radiotherapy combined with sorafenib has better efficacy in unresectable hepatocellular carcinoma: a systematic review and meta-analysis. Clin Exp Med (2023) 23(5):1537–49. doi: 10.1007/s10238-022-00972-4 PMC10460724 36495367

[39] Su K Huang W Li X Xu K Gu T Liu Y. Evaluation of lactate dehydrogenase and alkaline phosphatase as predictive biomarkers in the prognosis of hepatocellular carcinoma and development of a new nomogram. J Hepatocell Carcinoma (2023) 10:69–79. doi: 10.2147/JHC.S398632 36685113 PMC9850255

[40] Su K Shen Q Tong J Gu T Xu K Li H. Construction and validation of a nomogram for HBV-related hepatocellular carcinoma: A large, multicenter study. Ann Hepatol (2023) 28(4):101109. doi: 10.1016/j.aohep.2023.101109 37100384

[41] Bian Z Zhou M Cui K Yang F Cao Y Sun S. SNHG17 promotes colorectal tumorigenesis and metastasis *via* regulating Trim23-PES1 axis and miR-339-5p-FOSL2-SNHG17 positive feedback loop. J Exp Clin Cancer Res (2021) 40(1):360. doi: 10.1186/s13046-021-02162-8 34782005 PMC8591805

[42] Wang Y Liu J Ren F Chu Y Cui B. Identification and validation of a four-long non-coding RNA signature associated with immune infiltration and prognosis in colon cancer. Front Genet (2021) 12:671128. doi: 10.3389/fgene.2021.671128 34290738 PMC8287327

[43] Li Y Lu Y Chen Y. Long non-coding RNA SNHG16 affects cell proliferation and predicts a poor prognosis in patients with colorectal cancer via sponging miR-200a-3p. Biosci Rep (2019) 39(5):6. doi: 10.1042/BSR20182498 PMC6522740 30962265

[44] Xu M Zhang R Qiu J. A four immune-related long noncoding RNAs signature as predictors for cervical cancer. Hum Cell (2022) 35(1):348–59. doi: 10.1007/s13577-021-00654-5 34846702

