# Supplementary figures and images for: Comprehensive analysis of disulfidptosis-related lncRNA features for prognosis and immune landscape prediction in colorectal cancer
Source: Front Oncol. 2023 Dec 27;13:1287808. doi: 10.3389/fonc.2023.1287808 (PMC10783935; doi:10.3389/fonc.2023.1287808)

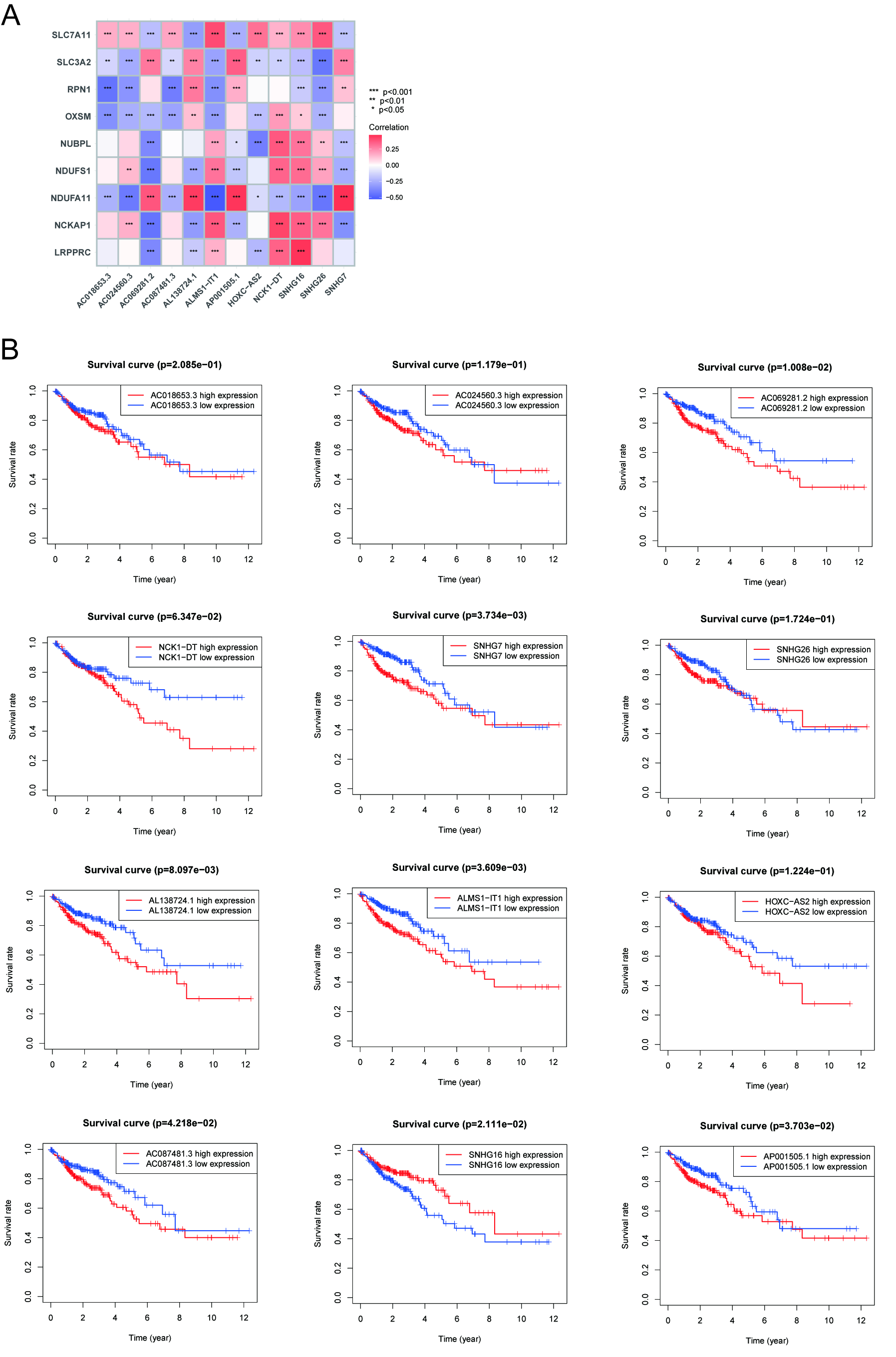

Supplement: Supplementary Figure 1 — (A) Correlation heatmap of the Disulfidptosis-Related Gene Set and Disulfidptosis-Related lncRNAs. DRGs, disulfidptosis-related genes; DRLs, disulfidptosis-related lncRNAs. (B) Survival analysis of the 12 Disulfidptosis-Related lncRNAs in Colorectal Cancer. [file Image_1.tif]
